# Superficial Skin Ulcers as an Initial Manifestation of Systemic Lupus Erythematosus

**DOI:** 10.7759/cureus.77701

**Published:** 2025-01-20

**Authors:** Juan Camilo Santacruz, Marta Juliana Mantilla, Sandra Pulido, Natalia Bocanegra-Oyola, Juan Diego Londoño, Carlos Agudelo

**Affiliations:** 1 Rheumatology Department, Spondyloarthropathies Research Group, Universidad de la Sabana, Chía, COL; 2 Rheumatology Department, Centro De Investigación En Reumatología Y Especialidades Médicas (CIREEM), Bogotá, COL; 3 Rheumatology Department, Centros Médicos Colsanitas, Bogotá, COL; 4 Dermatology Department, Universidad Del Rosario, Bogotá, COL; 5 Rheumatology Department, Universidad Nacional De Colombia, Bogotá, COL; 6 Rheumatology Department, Clínica Las Américas Auna, Medellín, COL

**Keywords:** leukocytoclastic vasculitis (lcv), skin ulcers, small vessel vasculitis, systemic lupus erythematosus, treatment choices

## Abstract

Cutaneous vasculitis is frequently present in patients with systemic lupus erythematosus (SLE), rheumatoid arthritis, and Sjögren's syndrome and is less common in dermatomyositis, systemic sclerosis, and relapsing polychondritis. Given the low prevalence of vasculitic involvement in this disease, the skin lesions described so far have had minimal semiological characterization. This has limited the provision of clear guidelines regarding their treatment, with most therapeutic interventions being extrapolated from idiopathic chronic leukocytoclastic vasculitis. We present the case of a female patient in her fifth decade of life who presented with superficial skin ulcers on the forearms as an initial manifestation, due to vasculitic involvement of small vessels secondary to SLE.

## Introduction

The clinical spectrum of vasculitis in the context of systemic lupus erythematosus (SLE) tends to be very broad and heterogeneous due to its great potential to compromise blood vessels of any size. However, the largest proportion of vasculitis in this entity is established in small vessels where dermatological manifestations are the main axis of its clinical manifestations [1]. The appearance of cutaneous vasculitis generally indicates an increase in systemic disease activity, both in SLE and in other autoimmune diseases. Patients with cutaneous vasculitis, compared to those without it, tend to have a longer disease duration, with an earlier onset and a higher prevalence in men [2]. They may also show p-ANCA (mostly) or c-ANCA by indirect immunofluorescence [3]. Additionally, there are no controlled clinical studies to help guide therapeutic decisions in this context and most of the evidence comes from case reports. Currently, little is known about the therapeutic outcomes of reported cases, but favorable clinical responses have been described with hydroxychloroquine, colchicine, mycophenolate, and rituximab [4-6].

## Case presentation

**Figure 1:**
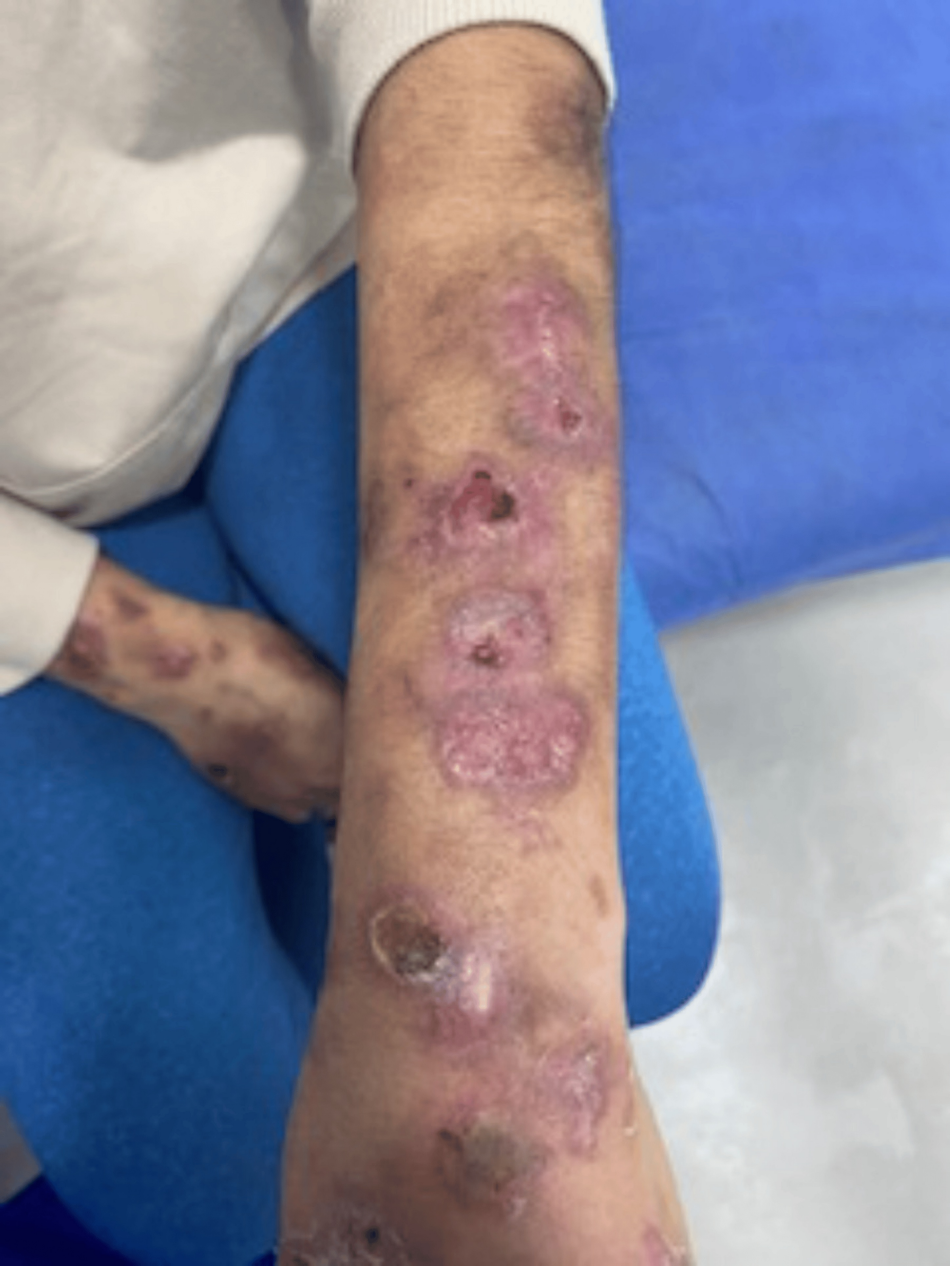
Skin lesions at the onset of clinical presentation Multiple cutaneous lesions are observed on the dorsum of the hand and the distal forearm, characterized by ulcers with hyperkeratotic crusts and irregular borders in certain areas. Additionally, several erythematous-violaceous macules and plaques are present, some of which exhibit focal areas of skin atrophy.

This is a 44-year-old female patient from San Juan de los Morros (Venezuela) who consulted for a seven-month history of localized ulcers on the upper extremities, mainly on the extensor surfaces of both forearms, which were purplish, painless, and diffuse in distribution. The initial diagnostic impression by dermatology was of generalized discoid lupus although the initial skin biopsy report described epidermal tissue with superfixation changes with thin epidermis, flattening of the network, ridges, and exostoses of polymorphonuclear cells, with a diagnosis suggestive of moderately differentiated squamous cell carcinoma (Figure 1). 

**Figure 2:**
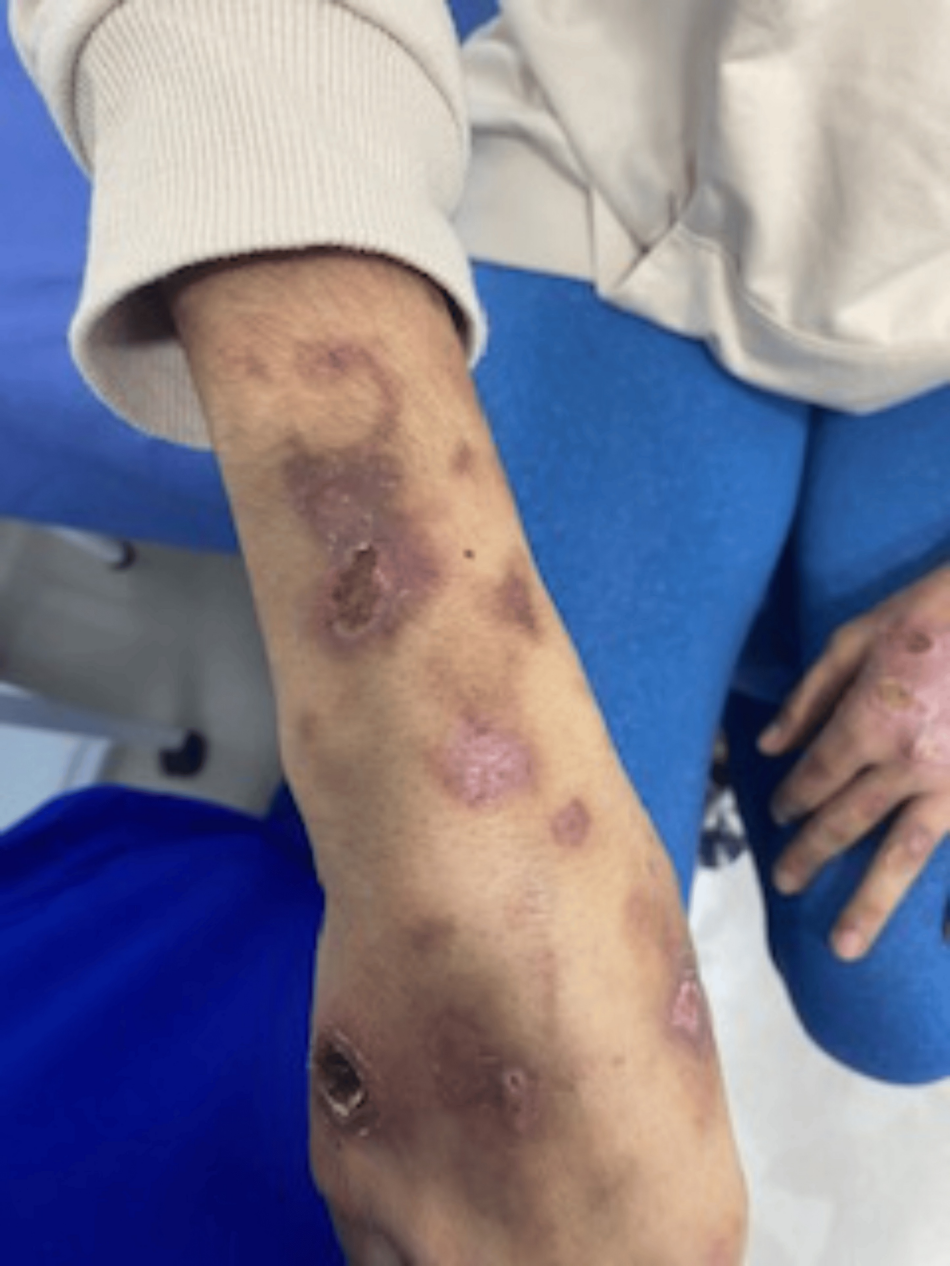
Evolution of skin lesions one month after treatment Some lesions exhibit persistent perilesional inflammation, while others display areas of scarring, reflecting varying stages of resolution and chronicity.

Initially, treatment was prescribed with 15 mg of oral methotrexate weekly and 1 mg of folic acid daily, but there was no clinical improvement. She subsequently attended a rheumatology consultation where she was referred to the hospital to determine whether it was a primary squamous cell carcinoma and assess its stage by taking CT scans. During hospitalization, the possibility of performing a new skin biopsy was evaluated, which showed microscopic characteristics similar to those previously observed; however, the pathologist's diagnostic impression was more oriented toward leukocytoclastic vasculitis. Further studies with complete immunology laboratories were indicated and the questioning was expanded due to the consideration of an autoimmune disease. The presence of alopecia was established by the review of systems at the same time as the appearance of the skin ulcers, but there was no photosensitivity, oral ulcers, or arthralgia. During the paraclinical tests conducted during hospitalization, the presence of proteinuria without hematuria was found on urinalysis, with no indication for a renal biopsy by nephrology. Positive findings included antinuclear antibodies at a titer of 1:1280 with a homogeneous pattern, positive extractable nuclear antigens (ENAs): SM: 28, RNP: 26, RO: 25, LA: 25 (reference value up to 25 IU/mL), positive anti-DNA at a titer of 1:320, marked hypocomplementemia, positive total ANCA for both anti-PR3 and MPO, and low-volume anemia, leading to the consideration of small vessel vasculitis as the initial manifestation of SLE. Treatment with prednisolone at 0.5 mg/kg of body weight and colchicine at 0.5 mg daily was initiated, with a slight improvement in some of the ulcers after completing the first month of treatment. However, the patient was referred back for hospitalization due to an outpatient report of 24-hour urine protein of 10 g, followed by loss of follow-up due to losing her affiliation with the mandatory health system (Figure 2). Below is a table describing the paraclinical studies performed during hospitalization (Table 1).

**Table 1:** Paraclinical tests performed during hospitalization ANCAS: antineutrophil cytoplasmic antibodies; ALT: alanine transaminase; AST: aspartate aminotransferase; ANAS: antinuclear antibodies; CRP: reactive c-protein; ENAS: extractable nuclear antigens; INR: international normalized ratio; RNP: ribonucleoprotein; SM: anti-Smith

Paraclinical studies	Initial result
Hemoglobin (gr/dL)	9.72
Hematocrit (%)	36.8
Mean corpuscular volume (fL)	71
Total leukocyte count (/µL)	12.640
Total neutrophil count (/µL)	9520
Total lymphocyte count (/µL)	2450
Total platelet count (/µL)	257.000
Creatinine (mg/dL)	1.09
Direct Coombs test	Negative
ALT U/L	12
AST U/L	9
ANAS (titers)	Positive 1/1280 (homogeneous pattern) AC-01
ENAS (U/mL)	Positive: SM: 28, RNP: 26, RO: 25, LA: 25 (reference value up to 25 U/mL)
Anti-DNA (UI/mL)	1/320 positive
C3 (mg/dL)	80
C4 (mg/dL)	10
Cardiolipin IgM (U/mL)	7.8 (positive)
ANCAS	Positive for both anti-PR3 and MPO (cut-off of 5 U/mL)
CRP (mg/dL)	-
Prothrombin time (sec)	44.9/14
Thromboplastin time (sec)	44.7/30
24-hour urine protein	268.4 mg/24 hours
Urinalysis	Sediment: red blood cells absent - proteins (+)

## Discussion

The prevalence of vasculitis in patients with SLE has had a great variability in different cohort studies, ranging from 11% to 36%. Although vasculitis in SLE can affect vessels of any size, small vessel vasculitis is the most common form of presentation [7]. The skin is one of the organs most frequently affected by lupus and the presence of cutaneous vasculitis has been identified in about 8% of patients with skin lesions associated with this disease [8]. In a European registry of 704 patients with SLE, the semiological characteristics of cutaneous vasculitis involvement are identified with great variability in its clinical presentations including petechiae, livedo reticularis, papulonodular lesions, palpable purpura, panniculitis, erythematous or necrotic plaques, astila hemorrhages, and superficial ulcers [9]. Urticarial and nodular lesions associated with ulcers have also been described, and in up to 29% of patients, there may be different types of lesions at the same time [10]. Cutaneous vasculitis has been described in 19-28% of patients with lupus, representing 89% of cases of vasculitis associated with this disease. Positive cryoglobulins, anti-RO antibodies, and antiphospholipid antibodies have been linked to an increased prevalence of cutaneous vasculitis in SLE [11,12]. The histopathological characteristics of small vessel vasculitis lesions in SLE are indistinguishable from those seen in other autoimmune diseases. Microscopic findings correspond to those of leukocytoclastic vasculitis. The main histological characteristics are polymorphonuclear infiltration in and around vessel walls, leukocytoclasia, tissue damage, and fibrinoid necrosis [13]. Although isolated descriptions exist, no case series or reports have comprehensively detailed the semiological characteristics of ulcers as an initial manifestation of SLE or evaluated the efficacy of current therapeutic approaches. In this case, the patient exhibited a partial response to glucocorticoids and colchicine. Laboratory findings included positivity for anti-ro antibodies and certain antiphospholipid antibodies; however, a complete profile, including cryoglobulins and rheumatoid factor, could not be obtained.

## Conclusions

Superficial skin ulcers as an initial manifestation of SLE represent an exceedingly rare presentation, attributed to the low prevalence of vasculitic involvement in this condition. The limited number of reported cases has left significant gaps in understanding their clinical presentation, response to currently available treatments, potential coexistence with other forms of vasculitis, and the optimal duration of therapy. Comprehensive documentation and analysis of such cases, including their therapeutic outcomes, are essential to establish standardized management protocols.
